# Study on Human Activity Recognition Using Semi-Supervised Active Transfer Learning

**DOI:** 10.3390/s21082760

**Published:** 2021-04-14

**Authors:** Seungmin Oh, Akm Ashiquzzaman, Dongsu Lee, Yeonggwang Kim, Jinsul Kim

**Affiliations:** Department of ICT Convergence System Engineering, Chonnam National University, Gwangju 61186, Korea; 198440@jnu.ac.kr (S.O.); zamanashiq3@chonnam.ac.kr (A.A.); 187761@jnu.ac.kr (D.L.); 206540@jnu.ac.kr (Y.K.)

**Keywords:** human activity recognition, active transfer learning, semi-supervised learning, semi-supervised active transfer learning, labeling reduction

## Abstract

In recent years, various studies have begun to use deep learning models to conduct research in the field of human activity recognition (HAR). However, there has been a severe lag in the absolute development of such models since training deep learning models require a lot of labeled data. In fields such as HAR, it is difficult to collect data and there are high costs and efforts involved in manual labeling. The existing methods rely heavily on manual data collection and proper labeling of the data, which is done by human administrators. This often results in the data gathering process often being slow and prone to human-biased labeling. To address these problems, we proposed a new solution for the existing data gathering methods by reducing the labeling tasks conducted on new data based by using the data learned through the semi-supervised active transfer learning method. This method achieved 95.9% performance while also reducing labeling compared to the random sampling or active transfer learning methods.

## 1. Introduction

Human activity recognition (HAR) technology is a field of research in which a person’s specific activity is recognized based on sensor data such as gyroscope and acceleration, camera images, and video data [1]. There are various studies on HAR that are currently being conducted. Recognizing human activity can be applied to surveillance systems that can detect health risks, safety, and emergency situations. For example, the number of caregivers who can care for elderly people living alone cannot keep up with the increase in the number of households living alone. This problem can be solved by detecting the behavior of the elderly living alone. HAR technology is used in various areas and it is also appropriate for applications in smart homes and health care services of the Fourth Industrial Revolution. Thus, HAR techniques have been continuously studied. Deep learning is a technology that trains machines that are not capable of conventional cognitive thinking to naturally recognize patterns using multiple processing layers without informing them of the data features through a neural network structure. The application of deep learning techniques has begun to produce better performance compared to methods used in existing studies, and have also begun to be applied naturally to HAR techniques. Most machine learning and deep learning are theoretically ill-informed for all activities, but they can achieve sufficient performance for labeled activity recognition with appropriate learning and models. Deep neural networks (DNNs) are models underlying various artificial intelligence-based models, and are machine learning algorithms that have been used in various studies. Therefore, various studies using deep neural networks have been conducted with aim of solving problems using less data or in different domains based on the knowledge held by the learned model. Transfer learning is a technology that can apply the solutions to new problems by utilizing or applying existing learned weights. However, deep learning requires a large number of data that must be labeled in order to be learned. In the field of HAR, there is the disadvantage that the individual sensors have to attach and act on their own. In addition, unlabeled data must be identified and labeled directly by the domain’s experts or administrators. As the data collected increases and becomes more diverse, the cost of these labeling tasks continues to rise. Active learning can be used to reduce the number of labeling tasks by asking the administrator to determine what data are needed to solve a problem. Active learning can solve problems in such a way that the deep learning models learn by judging the data that need labeling and requesting labeling for the most necessary of these data. Semi-supervised learning is a method of deriving performance by conducting first-order learning with small datasets where the labels exist as well as second-order learning, where there are large datasets without correct labels. This work proceeds with semi-supervised active learning, which combines semi-supervised learning with transfer learning and active learning to request labeling from administrators for the necessary data and no labeling if the labels can be fully predicted based on the previously learned data. While these technologies alone are not entirely novel, no study has examined the combination of the two techniques. This work addresses the idea of reducing the number of labeling requests to administrators through deep neural network models and transition models of learned deep neural networks, and also evaluates how using fewer labeling requests can lead to higher performance. We also propose and analyze new algorithms utilizing this idea in fields that are challenging and involve high costs for labeling tasks such as HAR. The rest of the paper proceeds as follows. Section 2 describes the previous work done in this field. Section 3 discusses the fundamentals of deep neural networks (DNN) and active learning (AL) as well as semi-supervised learning (semi-SL). Section 4 proposes our method and experiment. Section 5 assesses the performance according to the number of labels through evaluation metrics. Finally, Section 6 provides a summary of this work and future directions.

## 2. Related Research

### 2.1. Human Activity Recognition

In recent years, there have been many studies and approaches examining HAR. Finding patterns in HAR is complex and challenging, and is still an active area of research [2,3]. This section describes previous work on HAR using deep learning. HAR can be used in various fields such as smart health care, smart home, and elderly care. Wearable sensors, radio frequencies (RF), cameras, and sensors have been used to identify behavior. Advances in network technology have led to research on HAR data collection and activity recognition through the use of sensors. Lara, O. D. et al. had participants wear various wearable sensors, then recognized the human activity of these participants using external data such as environmental signals, location information, etc. [4]. As such, it is difficult to collect data in the field of human activity recognition as various data from wearables, cameras, and location information data must be merged for human activity recognition. In addition, video methods based on images have been used to address the problem of sensor attachment and portability [5]. Robertson, N. et al. developed a system for HAR in video sequences and derived rules for scenes through the hidden Markov model (HMM) [6]. However, given that attaching or holding wearable sensors can cause discomfort to humans, studies have examined how to naturally recognize activity using sensors mounted on devices such as smartphones [7,8]. In addition, San-Segundo, R. et al. also recognized human activity through HMM-based models using smartphones for HAR [9].

### 2.2. Deep Learning for Human Activity Recognition

Recently, the application of deep learning-based models in HAR [10,11,12] has been on the increase. Recent approaches in deep learning machine learning are mainly based on studies using deep neural network (DNN), long short-term memory (LSTM) [13], convolution neural network (CNN) [14] and others. As deep learning began to be studied in the field of activity recognition, Zhang, L. et al. combined HMM and DNN models to study human activity recognition [15]. Hassan, M. et al. extracted the characteristics of smartphone sensor data through kernel principal component analysis (KPCA) and linear discriminant analysis (LDA) and compared the performance of artificial neural network (ANN), support vector machine (SVM), and deep belief network (DBN) [16]; they showed that DBN performed the best for human behavior recognition. Wan, S. et al. compared SVM, CNN, LSTM, bidirectional LSTM, and MLP models for deep learning applications of human activity recognition, which led to the best performance of CNN models on the HCI-HAR dataset and PAMAP2 dataset [17]. Ullah, S. et al. presented a lightweight FCN-LSTM model using the HCI-HAR dataset [18]. These studies have only attempted to capture changes in sensors or changes in discriminant models for human activity recognition. Valuable data refers to data that can be used to produce good performance, even for smaller amounts. This can eventually lead to savings in labeling tasks. To this end, research is being conducted through various approaches.

### 2.3. Labeling Reduction Technologies

However, we typically collect data and then perform labeling tasks on all data. One study attempted to solve the labeling work to reduce the cost and effort required [19]. Active learning is one of the studies to solve this problem. Active learning is mainly studied to derive a high performance from a better number of methods for data sampling [20]. Tomanek, K. et al. reduced the effort for many sequence labeling tasks by 60% compared to random selection through semi-supervised active learning methods [21]. Liu, R. et al. sought to recognize human activity by learning the C4.5 decision tree classification model using acceleration sensor data from the human hip and wrists [22]; they also derived confidence levels for the data samples and compared the performance for both methods when confidence was both high and low. Bota, P. et al. conducted a study to predict human activity recognition by applying both semi-supervised learning and active learning for human activity recognition [23]. In addition, Stickic, M. et al. conducted comparisons through active and semi-supervised learning to reduce the labeling of PLCouple1 datasets for behavior recognition [24]. Gudur, G. K. et al. conducted a study applying active learning to reduce labeling of HAR data, resulting in good performance in reduced labeling [25]. However, deep learning models have yet to be applied to models for prediction. To this end, in this paper, we introduce semi-supervised active transfer learning based on a deep learning model to reduce labeling in the field of human activity recognition, wherein data labeling tasks are somewhat difficult.

## 3. Basic Theory for Labeling Reduction

### 3.1. Active Transfer Learning (ATL)

**Figure 1:**
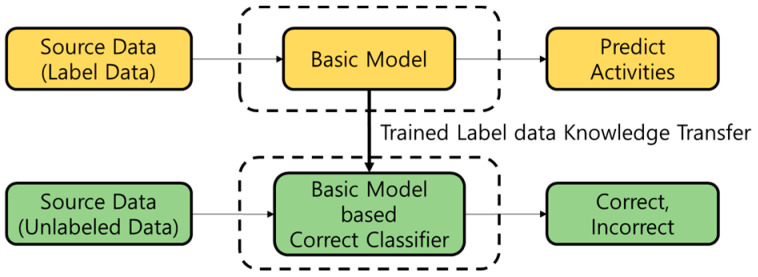
Transfer learning flow for labeling reduction.

Active learning (AL) is a technique in which a learned machine learning model selectively reviews unlabeled data for labeling tasks guided by humans. In practice, machine learning systems are trained with thousands or millions of data with human-processed labeling. However, machine learning procedures and performance can be made more accurate and efficient if humans only work with appropriate data to attach labels, and not all data. As such, AL performs the labeling work by sending a query requesting labeling to an administrator after sampling according to the rules or procedures based on a machine learning model, instead of humans labelling all the data. Data sampling that is used for active learning consists of two methods: diversity sampling and uncertain sampling. Uncertain sampling targets confusing data compared to the amount of information the current model has, and diversity sampling targets data that broadens the model’s knowledge. Transfer learning (TL), which is used for this, is not a newly created special technology for deep learning. Traditionally, traditional approaches were used to build and train machine learning models for each dataset. However, these approaches had the disadvantage of creating a new model if the dataset was insufficient or when the distribution of the data changed. Transfer learning is a method of utilizing the learned model to solve these problems. Comparing it to a person, a person who knows how to ride a bicycle can learn how to ride a motorcycle more easily than a person who cannot ride a bicycle. Transfer learning mainly consists of methods used with existing well-trained models that do not alter or fine-tune the learned weights. This allows trained models to benefit from extracting features, exploiting weights, and reducing learning time. As shown in Figure 1, ATL generates a correct classifier with information about the learning data based on the trained basic model and replaces the sampling scheme for active learning.

**Figure 2:**
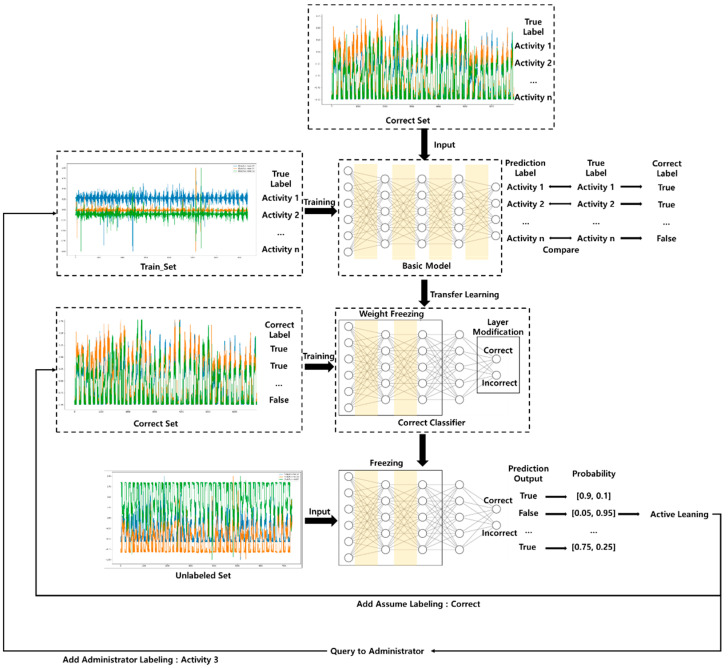
Active transfer learning architecture.

As shown in Figure 2, the active transfer learning flow consists of a machine learning model, transfer learning, and active learning [26]. Machine learning models such as the deep neural network (DNN) model or convolutional neural network (CNN) model can always be exploited. The DNN model learns to derive the desired output values by properly adjusting the weights according to the values entered in the input layer, and is based on the theory that the neural network can automatically understand a pattern of data. Many existing studies and literature have been used based on the underlying DNN theory. The DNN consists mainly of a fully connected layer and the entered value is calculated according to the weight of the node connecting each layer. The CNN model is used based on the theory that patterns can be understood and defined using the input matrix. CNN models consist of convolution and max pooling, etc., which extract visual information or low-dimensional features that appear in time series into high-dimensional features and utilize them to predict labels. Convolution is a sparse operation in which parameters are shared and reused. CNN are mathematical operations in which composite products can be applied to matrices.

### 3.2. Semi-Supervised Learning

**Figure 3:**
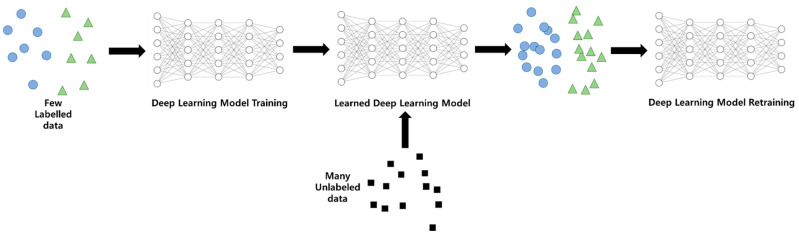
Semi-supervised learning architecture.

Deep learning techniques infer labels from numerical operations of input data values and model weight values to predict labels. Supervised learning (SL) is a technique used in various domains such as classification and regression by learning data and labels. SL learns and utilizes labeled data and is widely used for large classification and regression problems. Since early machine learning was used to classify certain data or predict values, supervised learning was a large axis of machine learning. However, there can be dozens to millions of learning data to train SL, and labels are essential for each data. Due to these problems, SL cannot be utilized without much adequate learning data. Semi-supervised learning (semi-SL) is a technique designed to compensate for these shortcomings. Based on the predicted values of the learned model, as shown in Figure 3, it is a technique that is used to label unlabeled data with simple rules such as threshold and to train machine learning models afresh with existing learning data to enhance performance

## 4. Proposed Methods

This section describes the details of the proposed method. We break down the proposed method into two main sections for illustration purposes. The first section contains the HAR dataset description. The second section deals with how to process the semi-supervised active transfer learning models.

### 4.1. Human Activity Recognition Dataset Description

Deep learning models have the disadvantage of low performance if they have inadequate learning and are not constructed with appropriate data. In this paper, we used the HCI-HAR dataset, which collected data using smartphones for human behavior recognition [7,27]. The dataset was collected among 30 volunteers aged between 19 to 48 years old, with each participant having a smartphone (Samsung Galaxy S2). Using the accelerometer and gyroscope built into the smartphone, three-axis linear acceleration and three-axis angular velocity were recorded at a speed of 50 Hz. The sensor data were pre-processed by the application of noise filters and then sampled in fixed-width sliding windows of 2.56 s and 50% overlap. The data contain 561 characteristics including the average, maximum, and minimum values; there are 10,299 data in total. The data are spread across six categories: *Walking*, *Walking_Upstairs*, *Walking_Downstairs*, *Sitting*, *Standing*, and *Laying.* The *Walking_Downstairs* data had the fewest labels (986), while the Laying data had the most labels (1407); there was an average of 1225 data for each label. However, the use of all 562 features of this dataset can include information that is too much learning for deep neural network models, and has the disadvantage of requiring a long time to learn. To address this, we extracted and used key features that determined the decision base using XGboost’s tree-based model [28].

Table 1 lists the parameters used to extract key features, while Table 2 presents the key feature names extracted. Previously, the decision tree using 562 features showed 90.2% accurate performance, while the decision tree using 50 features extracted showed 87.9% accurate performance, thus reducing the number of features by about 512 with only a 2.3% difference in performance.

To proceed in the experiment with a refined dataset using this process, we continued by splitting it into the same configuration as shown in Table 3.

The validation dataset derives predictive labels by inputting them to learned DNN-based models. We compared the derived predictive labels with the correct answers to compare whether or not the model fit the label, and we created a new correct dataset. To create the correct dataset, we created a dataset with ‘0′ if the actual and the predicted labels were similar, and ‘1′ if they were not similar. The correct dataset was then used to learn the transferred classifier that was correct.

### 4.2. Proposed Process

**Figure 4:**
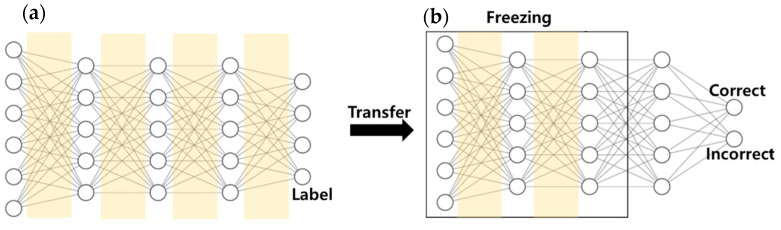
(**a**) DNN based basic model. (**b**) Transferred correct classifier model.

In this research, we constructed a DNN model as a base model for semi-supervised active transfer learning. The basic model consisted of four layers, where each layer consisted of (input size, 256), (256, 128), (128, 128), and (128, output size). The input size was 50 and the output size was 6, which represents the number of actions to be predicted. A ReLU activation function was used for each layer, and a drop-out technique was used to prevent overfitting. Semi-supervised active transfer learning consists of two main models, as shown in Figure 4. The DNN-based basic model learns the training dataset, and a transferred model-based correct classifier model transfers the basic model. Table 4 presents the details of the DNN-based basic model and the transferred correct classifier model, and weight freezing. Next, a DNN-based basic model learned from the existing configured train dataset. The learned basic model’s two-layer layers freeze weights and biases. The final layer is constructed to produce two output values (CORRECT, INCORRECT), and the transferred model learns the correct dataset. The unlabeled dataset enters this learned correct classifier model to verify the probability of the data, labels the data that have the highest probability for the labeling without queries, and adds the data with the lowest probability to the training dataset by querying the administrator. The experimental results from this study were derived using this process. Algorithm 1 illustrates the approach of semi-supervised active transfer learning.
**Algorithm 1.** Semi-Supervised Active Transfer Learning Algorithm**Input:** HAR Dataset ***BEGIN*** **Step 1:** Train the basic model with the training set **Step 2:** Create correct classifier that transfers the learned basic model **Step 3:** Input validation dataset into the learned basic model **Step 4:** Create a correct dataset according to the prediction of the basic model compared with the output and the actual value **Step 5:** Train the correct classifier model with the correct dataset (validation set) **Step 6:** Input the unlabeled dataset to the classifier to compare probability **Step 7:** Correct high probability data are sampled for semi-supervised learning **Step 8:** Incorrect high probability data are sampled for learning **Step 9:** Add sampled data to training set to retrain the basic model **Step 10:** Repeat the following process to efficiently label the unlabeled dataset to proceed with learning **END**

**Figure 5:**
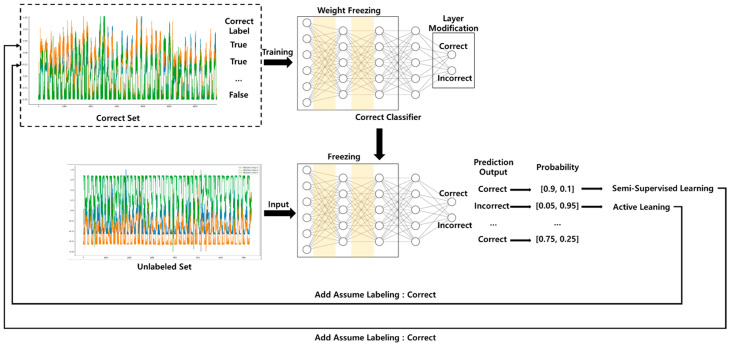
Correct classifier training of semi-supervised active transfer learning architecture.

**Figure 6:**
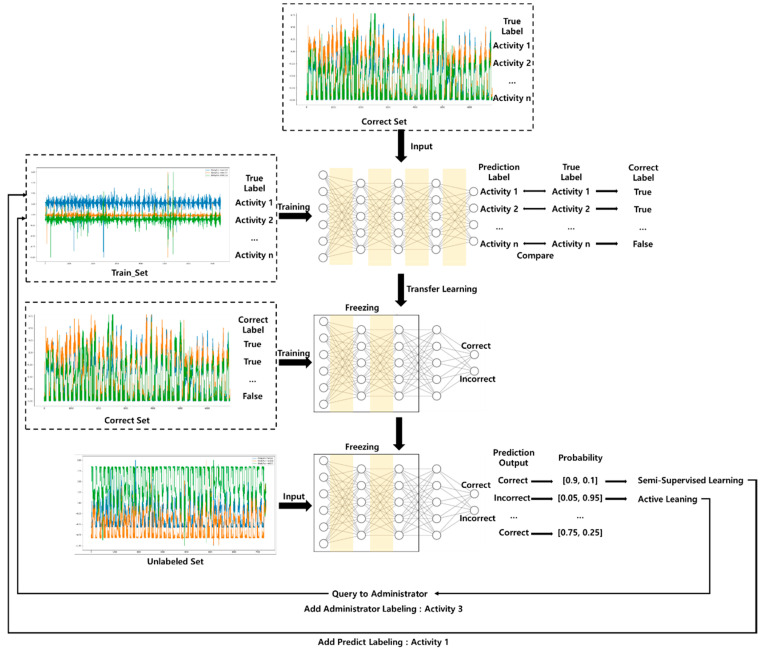
Training of semi-supervised active transfer learning architecture.

Figure 5 and Figure 6 illustrate the semi-supervised active transfer learning (SATL) that we propose in this paper. Figure 5 illustrates the tendency of the sampled data to reflect the trend of the sampled data by adding the correct classifier after sampling the data with the results derived through the model to perform the SATL. Figure 6 illustrates the data sampled through the semi-SL and AL methods of the entire flow of SATL, respectively, by requesting labeling from the administrator to obtain labels or adding them to the training set with the predicted labels. We produced machine learning models from learning data that had existing labeling such as the sequence of Algorithm 1. The trained model predicts activity based on the learned data by entering a validation dataset. We generated a correct dataset by comparing the actual and predicted values of the validation dataset. Next, we generated a transferred correct classifier based on weight-freezing and modification of the last layer of the learned machine learning model. We then learnt the correct classifier using the correct dataset. This allowed the algorithm to identify trends for unlabeled datasets. The correct classifier predicts either the correct or Incorrect label based on the unlabeled data input, which allows for a comparison of probability based on the knowledge of existing models. First, if the probability for correct is greater than 0.5, we can assume that we know the unlabeled data entered, but we hypothesized a threshold of 0.9 or higher because accuracy may be reduced early in the learning. If we have a figure above the threshold for correct, we can add a tendency by adding a dataset, assuming that it is correct in the correct dataset for semi-supervised learning. Subsequently, the sampled data for semi-supervised learning is added to the training set as the activity label predicted by the basic model. Second, probability for incorrect is sampled one by one in order of the highest. The data with the greatest probability that the correct classifier predicts as incorrect are added to the correct dataset by labeling it as correct, that is, assuming that the administrator receives a query and labels it as a real value. This process is repeated until there are no values predicted as incorrect. However, we initially restricted the underlying model to perform up to 100 active learning to solve the problem of insufficient information in order to reduce the value predicted by incorrect. We iterated this sampling to sample data that can provide the correct classifier with the maximum unlabeled dataset and added it to the training dataset to re-learn the underlying model.

## 5. The Performance According to the Number of Labeling

The neural networks used to implement the proposed method in this paper were developed using Python bases. Python makes it easy to implement networks by reducing the reliance on virtual environment configurations and libraries. Thus, the decentralized configuration of the Python-based environment can be useful in other real-world domains. The proposed DNN-based model and transferred model were constructed using the Pytorch library, which has a GPU-based optimization for parallel learning. The environment for this research was studied using high-performance server computers consisting of an Intel i9 X-series Processor, 128 GB RAM, Ubuntu 18.04, and NVIDIA GPU RTX 3090. The main parameters for the learning of all models consisted of 30 epochs, 30 batch sizes, drop out of 0.2, learning rates of 0.01, and early stopping of 15, and were trained with Adam optimization and cross entropy. The pre-processing of the dataset was done with regularization. We compared the random sampling performance, active transfer learning, and semi-supervised active transfer learning model with the number of learning data to assess the accuracy. Table 5 presents the number of data queries and maximum accuracy requested from the administrator, which show that the proposed method had a 0.3% lower performance than active transfer learning, while also reducing the number of queries for labeling requests by 12%.

**Figure 7:**
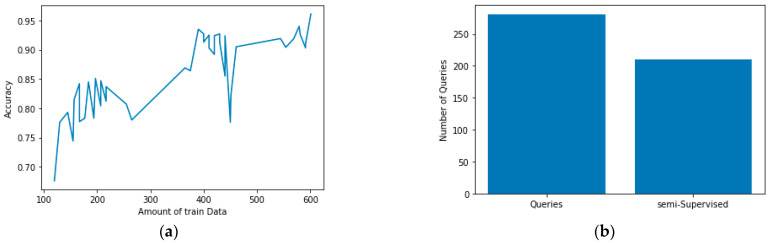
(**a**) Accuracy graph of convolutional neural network (CNN) based models; (**b**) Number of semi-supervised active transfer learning (SATL) queries for CNN based models.

We derived the performance of this SATL via the 1D CNN model using both the HCI-HAR data features as well as the DNN-based model. DNN-based models are heavily influenced by the number of features or amount of information in the existing data. The 1D CNN-based model is a model that recognizes behavior by deriving features over time based on data information. For the application of the 1D CNN model of HAR data, 561 datasets corresponding to five times were configured and used for learning. The configuration of the HAR dataset is different from the DNN-based model and is shown in Table 6. The initial learning data were tested for the performance of SATL with 400 fewer components than the DNN-based models. The CNN-based model was constructed as shown in Table 7. The training parameters of the CNN-based basic model and the CNN-based correct classifier model were 20 epochs, respectively, consisting of a 32 batch size, 0.01 learning rate, 0.5 dropout, and 15 early stopping. For optimization of the proposed model, we trained with Adam optimization and cross entropy. The performance of the model is shown in Figure 7a. We learned labeling data via 281 queries and 220 predicted labeling data using semi-supervised learning properties to derive a 96.1% accuracy. Figure 7b shows an accuracy graph of a CNN-based model and a predictive labeling data using 281 queries and 220 semi-supervised learning out of the 501 data learned.

In addition to the HCI-HAR dataset used in this paper, we validated the performance using the mHealth dataset [29]. The mHealth dataset consists of acceleration and gyro sensor data (23 pieces) attached to the chest, ankle, and arm with *Standing still*, *Sitting and relaxing*, *Lying down*, *Walking*, *Climbing stairs*, *Waist bends forward*, *Frontal elevation of arms*, *Knees bending*, *Cycling*, *Jogging*, *Running*, *Jump front & back* labeled data. The DNN-based model adds two linear full connected layers to add depth over the existing model. The dataset configuration for training DNN-based models that have learned mHealth datasets is the same as shown in Table 8 and the performance is represented in Table 9. The DNN-based model learned using active transfer learning was trained with 766 queries to derive 94.9% accuracy. However, the model using the proposed method learned 693 queries and 70 predicted labeling data, resulting in 95.9% performance.

## 6. Conclusions

The semi-supervised active transfer learning model proposed in this paper is a technique that uses existing semi-supervised learning, active learning, and transfer learning. Although it is not novel, no technology has previously applied it. Although the demand for data is increasing as research into deep learning continues expanding, labeling remains a challenging area as it is expensive. Labeling tasks are inevitably challenging, particularly in areas where humans need to collect data directly such as in HAR. To compensate for these shortcomings, the model used in the proposed work was able to guarantee 95.9% performance on the mHealth dataset while reducing the number of data labeling by 10% and the HCI-HAR dataset with 2.6% more accuracy and 80% less labeling than ATL. Therefore, the proposed semi-supervised active transfer learning is an effective way to reduce the cost of labeling tasks. The proposed research can be used in industries that require labeling tasks by administrators but need to effectively build data such as the medical field and the field of HAR.

## Figures and Tables

**Table 1:** Feature extraction with XGboost Tree-based decision model parameter.

Parameter	Value
Booster	gbtree
Scale pos weight	1
Learning rate	0.01
Col-sample by tree	0.4
Subsample	0.8
N estimators	200
Max depth	4
Gamma	10

**Table 2:** List of extracted key features.

Extracted Features			
*fBodyAcc-skewness()-X*	*tBodyGyro-sma()*	*tGravityAccMag-std()*	*fBodyAccJerk-bandsEnergy()-9,16.1*
*tGravityAcc-min()-X*	*fBodyAcc-std()-Z*	*angle(X,gravityMean)*	*fBodyAccJerk-bandsEnergy()-1,24.1*
*fBodyAccJerk-std()-Y*	*fBodyAcc-max()-X*	*fBodyAccMag-std()*	*fBodyAcc-bandsEnergy()-17,24.2*
*tGravityAcc-energy()-X*	*fBodyAcc-mad()-X*	*tGravityAcc-max()-X*	*fBodyAcc-bandsEnergy()-1,8.1*
*fBodyAcc-max()-Z*	*angle(Y,gravityMean)*	*tBodyAccMag-std()*	*fBodyAcc-bandsEnergy()-1,24*
*tBodyAcc-iqr()-X*	*tBodyAcc-mad()-X*	*tBodyGyroMag-sma()*	*fBodyAcc-bandsEnergy()-1,16.2*
*tBodyAcc-max()-X*	*tGravityAcc-mean()-Y*	*tBodyAccJerk-std()-X*	*fBodyAcc-bandsEnergy()-9,16.2*
*fBodyAcc-kurtosis()-X*	*tBodyAccJerk-mad()-Y*	*tBodyGyro-energy()-Z*	*fBodyAccJerk-bandsEnergy()-1,16.2*
*fBodyAccMag-mad()*	*tGravityAcc-max()-Y*	*tBodyGyroJerk-std()-X*	*fBodyAccJerk-bandsEnergy()-17,24.2*
*tGravityAcc-mean()-X*	*tBodyGyroJerk-sma()*	*tBodyAccJerk-max()-Y*	*fBodyAccJerk-bandsEnergy()-33,48.2*
*tGravityAcc-arCoeff()-Z,1*	*tBodyGyroJerkMag-mean()*	*tBodyGyroJerkMag-entropy()*	*fBodyAcc-bandsEnergy()-1,8.2*
*fBodyAccMag-energy()*	*tBodyGyroMag-mean()*	*fBodyBodyAccJerkMag-max()*	*fBodyGyro-bandsEnergy()-17,24*
*tBodyGyroJerk-mad()-X*	*tBodyGyroJerk-energy()-X*		

**Table 3:** Training/validation/testing/unlabeled separation statistics (DNN, HCI-HAR).

Total Data	Training Data	Validation Data	Testing Data	Unlabeled Data
10,299	500	1000	1000	7799

**Table 4:** Fully connected layer based basic model.

DNN Based Basic Model			Transferred Correct Classifier		
Layers	Output Shape	Weight Freeze	Layers	Output Shape	Weight Freeze
FC Layer (Linear)	50, 256	False	FC Layer (Linear)	50, 256	True
ReLU	50, 256	False	ReLU	50, 256	True
FC Layer (Linear)	256, 128	False	FC Layer	256, 128	True
ReLU	256, 128	False	ReLU	256, 128	True
Dropout	0.2	False	Dropout	0.2	False
FC Layer (Linear)	128, 128	False	FC Layer	128, 128	False
ReLU	128, 128	False	ReLU	128, 128	False
Dropout	0.2	False	Dropout	0.2	False
FC Layer (Linear)	128, 6	False	FC Layer	128, 2	False

**Table 5:** Comparison of the number of queries and maximum accuracy (DNN, HCI-HAR).

Random Sampling		Active Transfer Learning		Proposed Method	
Number of Queries	Accuracy	Number of Queries	Accuracy	Number of Queries	Accuracy
1000	92.9%	224	95.8%	198	95.5%

**Table 6:** Training/validation/testing/unlabeled separation statistics (CNN, HCI-HAR).

Total Data	Training Data	Validation Data	Testing Data	Unlabeled Data
10,239	100	1000	1000	8139

**Table 7:** Convolution neural networks based basic model.

DNN Based Basic Model			Transferred Correct Classifier		
Layers	Output Shape	Weight Freeze	Layers	Output Shape	Weight Freeze
1D CNN	5, 8, kernel_size = 5	False	1D CNN	5, 8, kernel_size = 5	True
ReLU	5, 8, kernel_size = 5	False	ReLU	5, 8, kernel_size = 5	True
1D CNN	8, 16, kernel_size = 5	False	1D CNN	8, 16, kernel_size = 5	True
ReLU	8, 16, kernel_size = 5	False	ReLU	8, 16, kernel_size = 5	True
1D CNN	16, 8, kernel_size = 5	False	1D CNN	16, 8, kernel_size = 5	True
ReLU	16, 8, kernel_size = 5	False	ReLU	16, 8, kernel_size = 5	True
Dropout	0.5	False	Dropout	0.5	False
MaxPooling1D	Kernel_size = 5	False	MaxPooling1D	Kernel_size = 5	False
FC Layer (Linear)	872, 100	False	FC Layer (Linear)	872, 100	False
ReLU	872, 100	False	ReLU	872, 100	False
FC Layer (Linear)	100, 6	False	FC Layer (Linear)	100, 2	False

**Table 8:** Training/validation/testing/unlabeled separation statistics (DNN, mHealth).

Total Data	Training Data	Validation Data	Testing Data	Unlabeled Data
16,384	1000	2000	2000	11,384

**Table 9:** Comparison of the number of queries and maximum accuracy (DNN, mHealth).

Active Transfer Learning		Proposed Method	
Number of Queries	Accuracy	Number of Queries	Accuracy
766	0.949%	693	0.959%

## Data Availability

Not applicable.
